# Co-Doped ErFeO_3_ for Dual-Band Laser Absorption with High-Temperature Stability

**DOI:** 10.3390/ma18081861

**Published:** 2025-04-18

**Authors:** Rui Liu, Linghao Pan, Fanqi Meng, Xia Feng, Qitu Zhang, Yi Hou, Lixi Wang

**Affiliations:** 1 College of Materials Science and Engineering, Nanjing Tech University, Nanjing 211816, China; 2 Jiangsu Collaborative Innovation Center for Advanced Inorganic Function Composites, Nanjing 211816, China

**Keywords:** dual-band laser absorption, high-temperature stability, co-doped ErFeO_3_, perovskites

## Abstract

The development of multi-band laser suppression materials has been driven by the limitations of single-band laser suppression materials. Inorganic ceramic materials, compared with organic laser suppression materials, photonic crystals, and metamaterials, offer significant advantages in fabrication methods and environmental stability. In this study, Co^3+^ ions, with relatively higher electronegativity, were introduced to substitute some Fe ion sites in ErFeO_3_. This substitution caused distortion in the crystal structure, reduced the unit cell volume, and altered the band structure. As a result, the band gap was reduced compared with that of ErFeO_3_, and the unique energy level transitions of Er ions were activated. This led to dual-band laser suppression with reflectances of 22.16% at 1064 nm and 35.63% at 1540 nm. Furthermore, after high-temperature testing at 1100 °C in air, the laser absorption performance could still be maintained with the intensity retention above 95%. This unique strategy for improving the band structure provides significant potential for applications in laser suppression.

## 1. Introduction

The development of multi-band compatible detection technology has become increasingly mature. Common laser detection primarily targets three main wavelengths: 1064 nm, 1540 nm, and 10.6 μm [1,2,3,4,5,6,7]. Research on single-band laser suppression materials has been relatively comprehensive. However, advances in laser detection technology necessitate the exploration of multi-band compatible laser suppression materials. The complex application scenarios of these materials demand environmental stability, particularly high-temperature stability [8,9]. Therefore, the development of multi-band compatible laser suppression materials must consider both performance and environmental durability.

Common laser suppression materials include organic materials [10], inorganic ceramic materials [11,12,13], photonic crystals, and metamaterials [14,15,16]. Organic laser suppression materials, such as cyanine dyes, exhibit good performance in the near-infrared region. However, their heat resistance needs improvement for high-temperature environments [17,18,19]. Inorganic ceramic materials offer better thermal stability but may lack the flexibility of organic materials. Photonic crystals and metamaterials are designed through artificial structuring, using various combinations of structures, sizes, and arrangements. These materials possess excellent laser suppression capabilities and superior structural design flexibility. Despite these advantages, their relatively poor environmental stability and complex fabrication processes present challenges for large-scale production [20,21].

Inorganic ceramic materials offer significant advantages, including simple fabrication processes, stable performance, and strong environmental durability. These materials can be prepared through straightforward methods such as solid-state and hydrothermal reactions. They maintain stable performance in high-temperature oxidative environments and exhibit long-term stability under harsh conditions, such as exposure to acids, bases, and salts [22,23,24]. For instance, Qin et al. [25] prepared Al/ATO materials that achieved 43.45% laser absorption at 1064 nm. Similarly, Hao et al. [26] developed Sm_2_O(CO_3_)_2_·xH_2_O, which also demonstrated good absorption at 1064 nm. This is mainly due to the fact that the rare-earth ion Sm produces a specific energy level jump, i.e., from the ground state energy level ^6^H_5/2_ to the excited state ^6^F_9/2_, when it is excited by the incident light, resulting in a characteristic absorption peak at 1064 nm. Despite these advancements, research on multi-band laser suppression using ceramic materials remains relatively limited. Zhu et al. [27] achieved 62.27% laser absorption at 1064 nm and 41.3% at 1540 nm by co-doping rare earth aluminates (Al_2_O_3_-Re_2_O_3_) with Sm^3+^ and Dy^3+^ ions. Xia et al. [28] employed electrospinning to prepare Er^3+^-doped SnO, achieving reflectances of 47.3% at 1064 nm and 42.1% at 1540 nm. These two absorption peaks correspond to the excited absorption of Er^3+^ ions from ground state energy level ^4^I_15/2_ to energy level ^4^I_11/2_ and energy level ^4^I_13/2_. However, existing multi-band laser suppression ceramic materials have drawbacks, such as poor absorption performance or insufficient high-temperature stability. The rare-earth ferrite (ReFeO_3_) matrix selected in this work has already found extensive applications in optical and electrical fields [29,30,31]. By specifically substituting the A site or B site in ReFeO_3_, the optical properties and band structure can be effectively improved, leading to excellent laser suppression performance and enhanced high-temperature stability [32,33,34]. By doping the B site of ReFeO_3_ with Co ions, the forbidden band width of the material can be effectively reduced, thus realizing the enhancement of the light absorption efficiency [29]. In addition, the doping of Co ions also reduces the cell volume of ReFeO_3_ to a certain extent, which changes the distance between rare earth ions and enhances the capacity transfer.

ErFeO_3_ belongs to a kind of rare-earth ferrate; for ErFeO_3_, the Er ions are located in the cavities formed by the octahedra, and the Fe ions are coordinated with six oxygens, which are located in the center of the FeO_6_ octahedra formed by the oxygen ions. ErFeO_3_ is allowed to be excited by light-interacting charge carriers due to its small bandgap, and this has led to its widespread use in photocatalysis and other research [35,36]. At this stage, relatively little research has been done on ErFeO_3_ for laser suppression. However, its small band gap and the unique energy level jumps of Er ions indicate some potential in laser suppression.

In this study, Co^3+^-doped ErFeO_3_ was prepared using a simple solid-state reaction. The substitution of Fe ions with Co^3+^ ions, which possessed higher electronegativity, improved the overall band gap structure of the material. This enhancement facilitated the transitions of internal electrons with Er ions, inducing characteristic absorptions at 1064 nm and 1540 nm. In addition, the samples after the high-temperature oxidation test at 1100 °C still retained more than 95% of the original performance of the absorption properties, indicating that our prepared materials had a certain high-temperature stability. By analyzing its morphology, internal structure, and spectral characteristics, the study revealed its potential applications in multi-band laser suppression.

## 2. Materials and Methods

### 2.1. Experimental Materials

The raw materials, including ferric oxide (Fe_2_O_3_), erbium oxide (Er_2_O_3_), and cobalt (III) oxide (Co_3_O_4_), were purchased from Macklin Chemistry Co., Ltd. (Shanghai, China), with all materials having a purity of over 99%. Anhydrous ethanol, used as a grinding and dispersing agent, was purchased from Aldrich Chemistry Co., Ltd. (Shanghai, China).

### 2.2. Synthesis of ErFe_(1−x)_Co_x_O_3_

ErFe_(1−x)_Co_x_O_3_ (x = 0, 0.01, 0.03, 0.05, and 0.07) samples, abbreviated as EFCO, were synthesized using conventional solid-state reaction methods. The raw materials were high-purity Er_2_O_3_, Fe_2_O_3_, and Co_3_O_4_, which were accurately weighed in stoichiometric units using a digital analytical balance. For example, for ErFe_0.95_Co_0.05_O_3_, which is ErFeO_3_ doped with 5 mol% Co, we weighed 0.01 moles of Er_2_O_3_, 0.0095 moles of Fe_2_O_3_, and 0.0005 moles of Co_3_O_4_. The weighed materials were then ground with a mortar and pestle in air and anhydrous ethanol for 2 h to ensure homogeneity. The resulting powder mixture was placed in an alumina crucible and heated from room temperature to 900 °C at a rate of 3 °C/min and held for 2 h. After initial calcination, the powder was ground again for 1 h to ensure homogeneity. The homogenized powder was then heated from room temperature to 1300 °C at a rate of 3 °C/min and held for 2 h. Finally, the obtained powder was ground for another hour to ensure its homogeneity. The final sample obtained was in powder form under macroscopic observation.

### 2.3. Characterization

The composition and phase of the samples were analyzed using an X-ray diffractometer (Smartlab 9KW, Nihon Rikaku Corporation, Tokyo, Japan) and Rietveld refinement, with X-ray diffraction scanning conducted over a range of 10° to 80°. Phase transitions were examined using a Fourier transform infrared spectrometer (Thermo Nicolet IS 5, Thermo Fisher Scientific, Waltham, MA, USA) and a thermogravimetric analyzer (STA449F3, Netzsch Instruments, Bavaria, Germany). The morphology and particle size distribution of the products were investigated using a field emission scanning electron microscope (FEI Scios 2 HiVac, Thermo Fisher Scientific, Waltham, MA, USA) and a transmission electron microscope (FEI-TALOS-F200X, Thermo Fisher Scientific, Waltham, MA, USA). Changes in the valence state of the samples were analyzed using a Raman spectrometer (Labram HR Evolution, Horiba Scientific, Kyoto, Japan) and X-ray photoelectron spectroscopy (Thermo Fisher Nexsa, Thermo Fisher Scientific, Waltham, MA, USA). Additionally, the optical spectra of the samples were measured using a UV-3600 UV-visible near-infrared spectrophotometer (Shimadzu Corporation, Kyoto, Japan).

## 3. Results and Discussion

### 3.1. Formation and Deformation of EFCO

**Figure 1:**
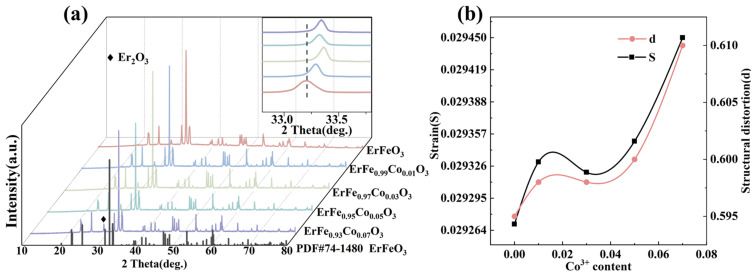
(**a**) X-ray diffraction patterns of EFCO at different doping levels. (**b**) Structural distortion factor *d* and orthorhombic strain factor *S* at different doping levels.

**Figure 2:**
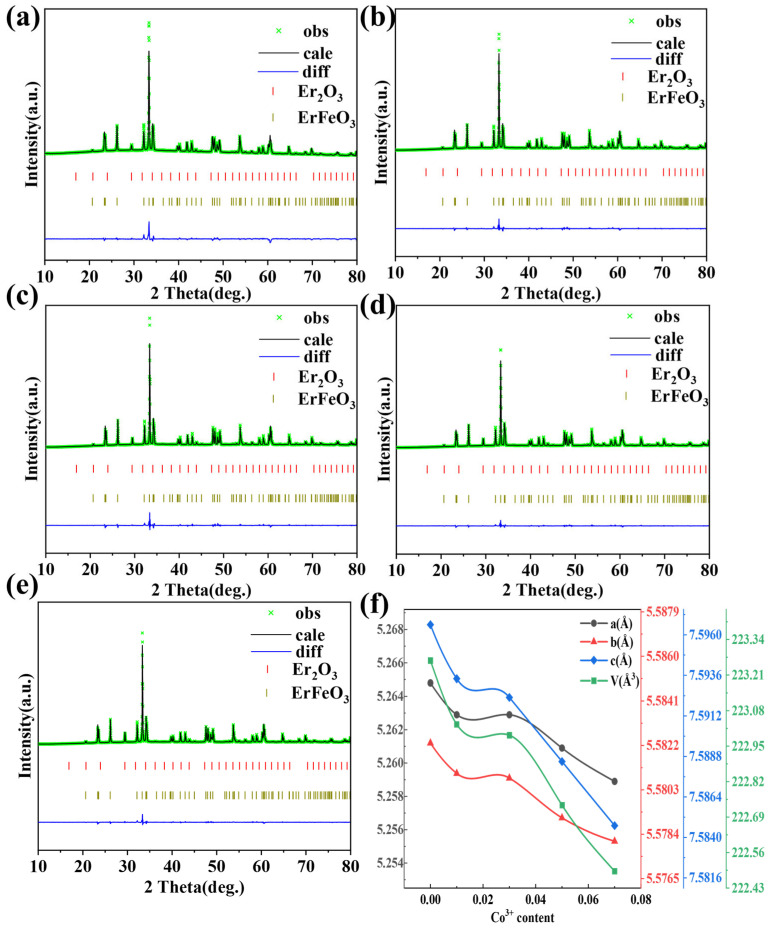
(**a**–**e**) Rietveld refined X-ray diffraction spectrum of EFCO. (**f**) Variation of lattice parameters and cell volume.

Figure 1a displays the X-ray diffraction (XRD) patterns for samples with varying levels of cobalt ion doping. The main peaks for all samples aligned with ErFeO_3_ (PDF#74-1480), hereafter referred to as EFO, and exhibited an orthorhombic perovskite structure with the Pbnm space group. The diffraction peak around 30° corresponded to Er_2_O_3_. Rietveld refinement analysis, conducted using GSAS software (SVN version 5789), determined the lattice parameters and unit cell volume, with a low R_wp_ (R_wp_ < 15%) indicating reliable results [37]. These results are summarized in Table 1. The lattice parameters and unit cell volume decreased as the Co ion doping concentration increased. This trend was attributed to the smaller ionic radius of Co^3+^ (0.61 Å) compared with Fe^3+^ (0.645 Å) [38]. The XRD patterns also revealed that the strongest diffraction peak (112) shifted toward higher angles with increasing Co ion doping concentration. According to Bragg’s diffraction equation 2dsinθ=nλ, this shift indicated a reduction in unit cell parameters, confirming successful Co ion doping and alteration of the unit cell parameters. The changes in structural parameters obtained from Rietveld refinement further corroborated this result, as shown in Figure 2f. In rare earth ferrites (ReFeO_3_), Re^3+^ ions typically occupy large cavities formed by octahedra, leading to orthorhombic distortion. According to Glazer’s study [39], these distortions arise from three primary factors: (1) tilting of the anion octahedra (O(1) and O(2)), (2) displacement of rare earth ions, and (3) distortion of FeO_6_ octahedra. Table 1 also presents the bond lengths and angles for Fe-O and Fe-O-Fe. In the entire EFCO structure, Fe^3+^ ions were octahedrally coordinated. Compared with undoped EFO samples, increasing Co ion doping in EFCO samples resulted in a gradual decrease in the Fe-O(1)-Fe bond angle, a gradual increase in the Fe-O(2)-Fe bond angle, and a decrease in the Fe-O bond length. These changes indicated that Co ion doping enhanced the distortion of the EFCO structure.

To further investigate the relationship between increased Co ion content and FeO_6_ octahedral distortion in the EFCO system, we quantitatively analyzed the structural distortion factor *d* and the orthorhombic strain factor *S*. The formulas used for these calculations are provided in references [40,41]. The results of these analyses are presented in Table 1. The structural distortion factor *d* describes the deformation of the unit cell. In contrast, the orthorhombic strain factor *S* characterizes the distortion of the octahedra within the orthorhombic unit cell:

(1) $$S=2\frac{b-a}{b+a}$$

(2) $$d=\frac{{\left(\frac{a}{\sqrt{2}}-a_{p}\right)}^{2}+{\left(\frac{b}{\sqrt{2}}-a_{p}\right)}^{2}+{\left(\frac{c}{2}-a_{p}\right)}^{2}}{3{a_{p}}^{2}}\times 10^{4}$$

(3) $$a_{p}=\frac{1}{3}\left(\frac{a}{\sqrt{2}}+\frac{b}{\sqrt{2}}+\frac{c}{2}\right)$$

The calculated values of *S* and *d* both gradually increased with the rise in Co doping concentration, as shown in Figure 1b. This suggested that Co ion doping enhanced the distortion of the FeO_6_ octahedra. In EFCO, the compression of unit cell parameters resulted from slight FeO_6_ octahedral distortion due to differences in ionic radii. The increased strain and structural distortion were attributed to changes in ion size and were clearly related to changes in the structural parameters. In EFCO, the ionic radius of Er^3+^ was smaller compared with the preceding rare earth ions. This caused the symmetric perovskite structure of FeO_6_ octahedra and the dodecahedral symmetry of the R-O_12_ framework to tilt or bend toward the R^3+^ site to reduce excess space. Consequently, this decreased the lattice parameters and reduced the structural symmetry, resulting in additional diffraction peaks in the X-ray diffraction patterns. This phenomenon explained the observed Er_2_O_3_ diffraction peaks [42]. The reported experimental results indicated that the cooperative rotation and tilting of FeO_6_ octahedra were closely related to the magnetic and optical properties of ReFeO_3_. It is anticipated that the substitution of Co ions will enhance the optical properties of EFO [34,43].

**Figure 3:**
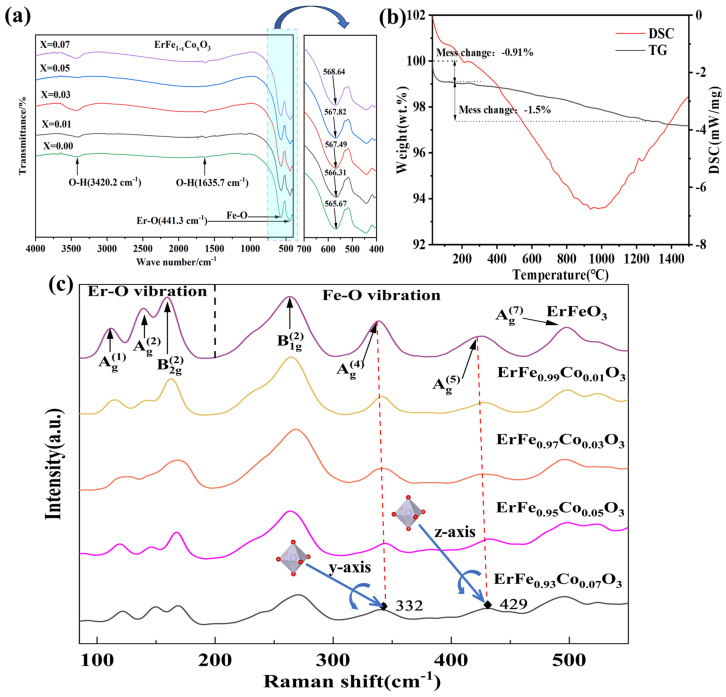
(**a**) FT-IR spectra of ErFe_1−x_Co_x_O_3_ (0 ≤ x ≤ 0.07) samples. (**b**) TG–DSC curve of the synthesized EFCO sample. (**c**) Raman spectra of EFCO (x = 0–0.07).

At the micrometer or nanometer scale, molecular vibrations induced by infrared radiation absorption were crucial for identifying functional groups in the prepared samples. Each functional group corresponded to a specific wavenumber range of infrared radiation, resulting in a unique fingerprint spectrum for each sample [44]. Figure 3a shows the FT-IR spectra of EFCO samples with varying Co^3+^ ion doping levels. Characteristic spectral bands, corresponding to specific infrared-active vibrational modes, were observed in the wavenumber range of 400 cm^−1^ to 4000 cm^−1^. Notably, the band at 441.3 cm^−1^ corresponded to the characteristic vibration of Er-O, and the band at 565.67 cm^−1^ corresponded to the characteristic stretching vibration of Fe-O [45,46]. As the Co^3+^ ion doping level increased, the stretching vibration peak of Fe-O shifted progressively toward higher wavenumbers. This change was likely caused by unit cell contraction due to the doping of Co^3+^ ions with smaller ionic radii. The unit cell contraction increased the force constant of the vibrational bonds, resulting in a shift of the peak position to higher wavenumbers. This phenomenon is also shown in the remaining studies of Co-doped ortho-ferrites [29,34]. The broad absorption peaks at 3420.2 cm^−1^ and 1635.7 cm^−1^ were attributed to the coupling effect of O–H bond stretching vibrations [47]. The presence and changes of these spectral bands further confirmed the successful synthesis of the EFCO samples.

The TG–DSC curve of the precursor is shown in Figure 3b. The TG curve revealed two main stages of weight loss. The first stage occurred between 20 °C and 157.68 °C, accounting for 0.91% of the weight loss, and was attributed to the removal of adsorbed water generated during ethanol grinding. The second stage occurred between 157.68 °C and 1248.29 °C, accounting for 1.5% of the weight loss. This stage was accompanied by a broad exothermic peak in the DSC curve. The weight loss at this stage was mainly due to the decomposition of the precursor feedstock and subsequent reactions between the decomposed materials leading to the formation of EFCO crystals. In addition, the Co ion doping process was accompanied by a localized release of lattice oxygen, leading to the formation of oxygen vacancies, which also contributed to the mass loss. This observation aligned with descriptions in the literature [48]. After 1250 °C, the weight loss stabilized, indicating that the selected temperature of 1300 °C in the experiment was sufficient to produce the desired EFCO.

The structural distortion caused by the doping of Co ions was closely related to the lattice vibration frequency. Figure 3c presents the Raman spectra of EFCO at various doping levels. Previous studies have demonstrated that the phonon modes of ReFeO3 in the Brillouin zone can be described by Equation (4) [49]. Among these phonon modes, 24 were Raman-active vibrations, 28 were infrared-active vibrations, and the remaining 8 were inactive modes [50].

(4) $$\Gamma {=7A}_{g}+8A_{1u}{+7B}_{1g}{+8B}_{1u}{+5B}_{2g}{+10B}_{2u}{+5B}_{3g}{+10B}_{3u}$$

In our measurements, we observed only seven Raman vibration modes (5A_g_ + 1B_1g_ + 1B_2g_), which was significantly fewer than the number reported in the literature. This discrepancy may be due to the overlapping of Raman vibration bands or limitations of the instrumentation. The specific mode assignments are detailed in Table 2. Peaks below 200 cm^−1^ were primarily attributed to the stretching vibrations of Er^3+^ and O^2−^, while peaks above 200 cm^−1^ were associated with the vibrations of O^2−^ or the FeO_6_ octahedra. The Raman vibration bands at 116 cm^−1^, 141 cm^−1^, 332 cm^−1^, 429 cm^−1^, and 501 cm^−1^ corresponded to the A_g_ modes. The bands at 163 cm^−1^ and 263 cm^−1^ were assigned to the B_2g_ and B_1g_ modes, respectively. Notably, the mode at 332 cm^−1^ was attributed to the rotation of the FeO_6_ octahedra around the y-axis, while the A_g_^(5)^ mode at 429 cm^−1^ was due to the rotation of the FeO_6_ octahedra around the z-axis.

From Figure 3c, we observed that as the doping concentration increased, the Raman peak positions shifted gradually toward higher wavenumbers (corresponding to shorter wavelengths). This phenomenon, known as a “blue shift”, was primarily attributed to compressive strain in the sample. Additionally, this blue shift indicated a reduction in the Fe–O bond length, which was consistent with the bond length changes obtained through Rietveld refinement [52]. The broadening of the Raman peaks may be due to the formation of oxygen vacancies induced by doping elements in the perovskite structure. The intensities of the Raman modes were dependent on the movement of oxygen ions [53]. Existing studies have shown that Co^3+^ ions can be partially reduced to Co^2+^ ions. To maintain charge neutrality, partial oxygen vacancies and Fe^4+^ were generated in the structure, consistent with findings from XPS.

The elemental chemical valence states of the EFCO (x = 0.05) sample surface were analyzed using X-ray photoelectron spectroscopy (XPS). The high-resolution spectra of Fe 2p, Co 2p, and Er 4d are shown in Figure S1, with fitting results presented in Table S1. All results were calibrated with respect to the standard peak of C 1s at a binding energy of 284.8 eV. Figure S1a displays the high-resolution spectrum of Fe 2p. The peaks at 710.3 eV and 712.36 eV corresponded to Fe^3+^ 2p_3/2_ and Fe^4+^ 2p_3/2_, respectively, while the peak at 724.6 eV was assigned to Fe^3+^ 2p_1/2_. Additionally, the peaks at 717.77 eV and 731.99 eV were attributed to Fe^3+^ satellite peaks, indicating that Fe^3+^ was the predominant oxidation state in EFCO [54]. Figure S1b shows the high-resolution spectrum of Co. The primary peaks were at 780.58 eV (Co^3+^ 2p_3/2_) and 795.88 eV (Co^3+^ 2p_1/2_), with a peak at 782.82 eV attributable to Co^2+^. This indicated that Co ions in EFCO predominantly existed in the Co^2+^ and Co^3+^ oxidation states [55,56]. The relative areas of the XPS peaks represented the abundance of elements in the system. As shown in Table S1, the area of the Fe^3+^ peaks was significantly larger than that of the Fe^4+^ peaks, confirming that Fe^3+^ was the predominant oxidation state in the structure. The coexistence of Fe^3+^ and Fe^4+^ confirmed the successful doping of Co into EFO, which was consistent with the XRD results. In an air atmosphere, some oxygen molecules were adsorbed on the EFCO surface. Some Fe^3+^ in EFCO, acting as electron donors to the adsorbed oxygen, was oxidized to Fe^4+^.

Figure S1c presents the high-resolution spectrum of Er 4d, where the binding energy at 167.58 eV was characteristic of Er 4d. This finding indicated that Er ions in EFCO were in the trivalent state, consistent with descriptions in the literature [57,58]. In Figure S1d, the high-resolution spectrum of oxygen was divided into two peaks. The peak around 528.78 eV was attributed to lattice oxygen, while the peak around 531.02 eV corresponded to oxygen vacancies. Oxygen vacancies are inherent defects in perovskite materials, typically generated by oxygen atoms under high-temperature reactions. During high-temperature calcination, the introduction of divalent cobalt into EFO also resulted in oxygen vacancies to maintain charge neutrality. These oxygen vacancies facilitated electron transfer, accelerating the redox cycling of metal ions and leading to faster transitions between different oxidation states.

**Figure 4:**
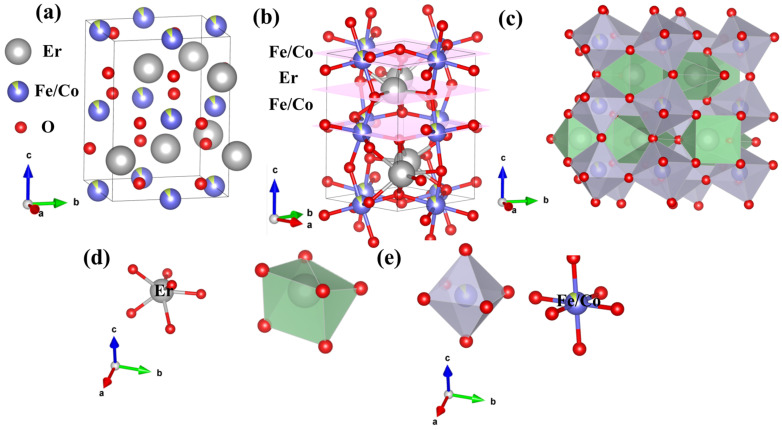
(**a**) Schematic diagram of the EFCO unit cell. (**b**) Periodic arrangement structure. (**c**) Three-dimensional structure of EFCO. (**d**) Er-O polyhedron. (**e**) Schematic diagram of FeO_6_ octahedra.

Figure 4a–e presents the structural diagram of EFCO, revealing that it adopted a classic distorted perovskite structure. The unit cell of EFCO contained four molecules. In this structure, Fe ions formed an octahedral configuration with six O ions, which further connected to create a three-dimensional network (Figure 4b,c). Er ions were located in the interstitial sites. Due to distortion effects, the Fe–O–Fe bond angles deviated from the ideal 180°. This distortion caused the FeO_6_ octahedra to tilt away from the c-axis, resulting in changes to the Fe–O–Fe bond angles. In Figure 4e, the O ions bonded to Fe occupy two distinct crystallographic sites, denoted as O1 and O2. O1 occupied two vertices of the octahedron along the [001] direction, while O2 occupied the remaining four vertices perpendicular to the [001] direction. This was consistent with the results obtained from Rietveld refinement. Additionally, in EFCO, Er ions were coordinated with six oxygen ions to form a polyhedron, as shown in Figure 4d.

**Figure 5:**
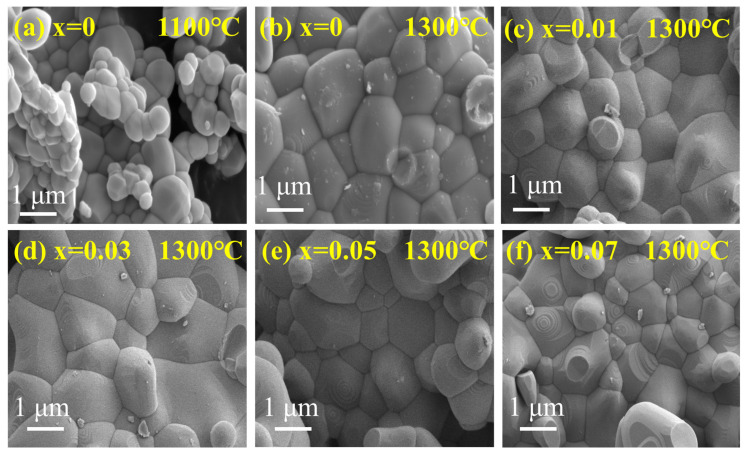
(**a**,**b**) SEM images of ErFeO_3_ at different calcination temperatures. (**c**–**f**) SEM images of EFCO (x = 0.01, 0.03, 0.05, and 0.07).

To investigate the microscopic structure of EFCO, Figure 5a–f presents SEM images of EFCO at various calcination temperatures and doping ratios. When the calcination temperature was below 1300 °C, the grains were underdeveloped, and significant voids were present. This resulted in a non-dense structure. As the calcination temperature increased, the grain size grew significantly and adopted a polygonal structure. This indicated more complete grain growth and reduced inter-particle voids, leading to a denser structure.

SEM images revealed that the particle size of EFCO decreased as the Co doping level increased. The average particle size distribution histograms, shown in Figure S2a–e, were obtained from Nano calculations. Initially, the average particle size of undoped EFO was 1.17 μm. With the doping of Co ions, this size decreased to 0.87 μm. This reduction in grain size could be attributed to the smaller ionic radius of Co^3+^ (0.61 Å) compared with Fe^3+^ (0.645 Å). As Co^3+^ partially replaced Fe^3+^ at the B site, the crystal contracted slightly to maintain structural stability, resulting in smaller grain sizes. This trend aligned with findings reported in the literature [59].

**Figure 6:**
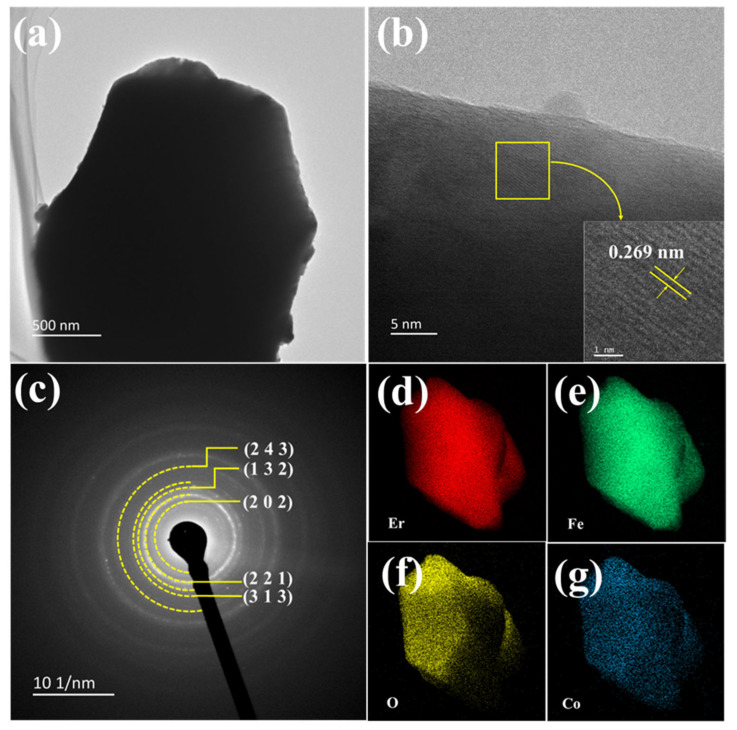
(**a**) TEM image of EFCO (x = 0.05). (**b**) HRTEM image of EFCO (x = 0.05). (**c**) SAED image of EFCO (x = 0.05). (**d**–**g**) EDS images of Er, Fe, O, and Co elements.

To further investigate the internal structure of EFCO crystals, Figure 6 presents TEM images of EFCO (x = 0.05) observed at different magnifications. The HRTEM images revealed clear lattice fringes of EFCO particles. The (112) crystal plane was observed with a plane spacing of 0.269 nm, consistent with the PDF card (PDF#74-1480). Figure 6c shows the SAED pattern of the selected area. The yellow rings correspond to the polycrystalline diffraction rings produced by reflections from the (243), (132), (202), (221), and (313) crystal planes, matching the expected positions. Figure 6d–g display the elemental distribution maps of EFCO (x = 0.05). The distribution of Er, Fe, and O elements was dense and uniform, indicating that these elements were major components of EFCO. In contrast, the distribution of Co ions was more sparse, confirming the successful doping of Co ions into the system. The EDS elemental distribution ratios in Figure S3 also supported this conclusion.

### 3.2. Optical Properties Analysis

For laser suppression materials, it is crucial that they exhibit characteristic absorption capabilities at specific wavelengths: 1064 nm, 1540 nm, or 10.6 μm, depending on the laser specifications. The EFCO material developed in this study demonstrated dual-band characteristic absorption at 1064 nm and 1540 nm. To evaluate its laser suppression performance, we examined optical parameters including reflectance spectra, absorption spectra, band gap, and threshold wavelength.

**Figure 7:**
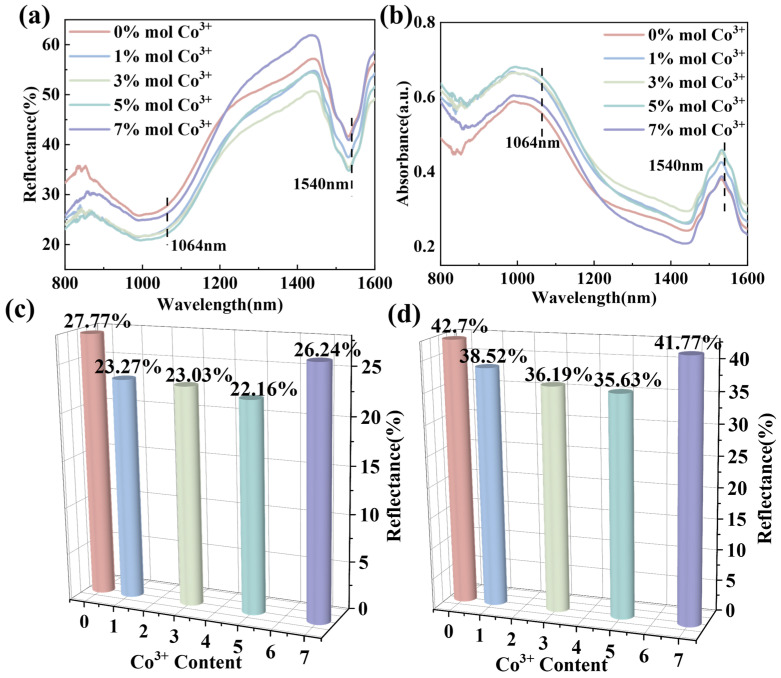
(**a**) Comparison of reflectance at different Co^3+^ doping concentrations. (**b**) Absorption spectra at different Co^3+^ doping concentrations. (**c**) Detailed reflectance of EFCO at 1064 nm under different x values. (**d**) Detailed reflectance of EFCO at 1540 nm under different x values.

Figure 7a,b present the diffuse reflectance and absorption spectra of EFCO samples at varying doping concentrations. The spectra exhibited distinct characteristic absorption peaks at 1064 nm and 1540 nm. The absorption effect intensified with increasing Co ion doping, peaking at 5% Co doping. At this concentration, EFCO (x = 0.05) showed reflectance values of 22.16% at 1064 nm and 35.63% at 1540 nm. When Er^3+^ ions were excited by light, they transitioned from the ground state level ^4^I_15/2_ to the excited state level ^4^I_11/2_. The energy required for this transition was close to that of 980 nm light, resulting in a broad characteristic absorption peak near 1064 nm. The absorption peak at 1540 nm was attributed to the transition of Er^3+^ ions from the ground state ^4^I_15/2_ to the higher energy level ^4^I_13/2_ upon absorbing incident photon energy. After transitioning to higher energy levels, electrons typically returned to the ground state through radiative or non-radiative transitions. Alternatively, excess energy may be transferred to other ions via non-radiative energy transfer. In some instances, Er^3+^ ions in the metastable energy level underwent downward transitions to produce amplified spontaneous emission and may also have transitioned to a higher energy level ^4^I_9/2_, leading to absorption. The emission spectrum from ^4^I_13/2_ to ^4^I_15/2_ overlapped with the absorption spectrum from ^4^I_13/2_ to ^4^I_9/2_ [60]. The energy involved in this process was approximately equivalent to the energy of light at a wavelength of 1540 nm. Therefore, EFCO material can be considered an excellent dual-band compatible laser suppression material. Figure S4 compares the reflectance performance of EFCO at 1100 °C under different oxidation times. The data indicated that EFCO maintained excellent laser rejection performance in high-temperature environments. Specifically, the absorption performance of EFCO remained at 95% after high-temperature oxidation at 1540 nm and 96% at 1064 nm, highlighting its potential for application in high-temperature settings.

Examining the optoelectronic properties of EFCO was crucial for understanding its laser suppression performance. We investigated changes in optical parameters, including the band gap, using various methods. These methods included the Kubelka–Munk formula (Formula 5) [61], empirical formulas, VB-XPS analysis, and threshold wavelength measurements. The Kubelka–Munk formula, in particular, evaluated changes in the optical band gap through reflectance measurements. This approach provided insights into the variations of localized states within the band gap.

(5) $$\left(F\left(R\right)h\nu \right)=A{\left(h\nu -E_{g}\right)}^{n}$$

**Figure 8:**
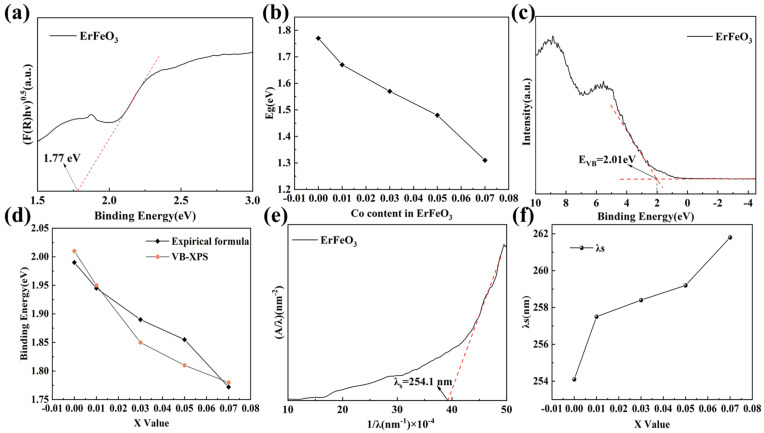
(**a**) Optical bandgap estimation of EFO. (**b**) Bandgap variation with different Co^3+^ doping levels. (**c**,**d**) Different estimation methods for the valence band. (**e**,**f**) Threshold wavelengths of EFO and EFCO.

In this analysis, R represents the reflectance, h denotes Planck’s constant, and *ν* is the frequency of light. E_g_ signifies the band gap energy. Figure 8a illustrates the estimated optical band gap of EFO (refer to Figure S5 for detailed data). By fitting the linear region of the graph and extending the tangent, the intercept on the X-axis corresponded to the band gap energy. This method determined the optical band gap of EFO to be 1.77 eV, aligning with values reported in the literature [62]. Figure 8b shows that the optical band gap of EFCO decreased with increasing Co^3+^ doping concentration, resulting in a 25.9% reduction compared with ErFeO_3_. This indicated that Co ion doping effectively tuned the band gap of EFO. The underlying mechanism involved the role of Fe at the B site in ReFeO_3_, which significantly influenced the band gap. Replacing Fe (electronegativity of 4.06) with Co (higher electronegativity of 4.3) increased the electronic bandwidth of the system, thereby reducing the band gap value [63,64]. Furthermore, the reduction in the band gap suggested the formation of localized states within the material. These localized states typically indicated the presence of oxygen vacancies, consistent with XPS analysis results [65]. A smaller band gap facilitated electronic transitions, making it easier for Er^3+^ ions to undergo excitation when irradiated with lasers at 1064 nm and 1540 nm. Consequently, this led to a reduction in the material’s reflectance.

Further analysis of the conduction band and valence band of EFCO was conducted using empirical formulas (Formulas (6) and (7)) and VB-XPS. The empirical formulas were as follows [63,66]. In these formulas, X represents the geometric mean of the absolute electronegativities of the atoms in EFCO. E_c_ is a constant of 4.5 eV relative to the standard hydrogen electrode. E_g_ denotes the band gap energy. E_CB_ and E_VB_ represent the conduction band and valence band, respectively.

(6) $$E_{CB}=X-E_{C}-\frac{1}{2}E_{g}$$

(7) $$E_{VB}=E_{CB}+E_{g}$$

Figure 8c presents the VB-XPS valence state spectrum of EFO. By fitting the linear portion with a tangent and extending it to intersect with the horizontal section below 0 eV, the valence band of EFO was determined to be 2.01 eV. Figure 8d shows the valence band positions obtained from VB-XPS and empirical formulas for different Co ion doping concentrations. The discrepancy between the two methods was small (refer to Figure S6 for detailed data). Additionally, as the Co ion doping concentration increased, the valence band continuously decreased, leading to a reduction in the band gap. This trend was consistent with the results obtained using the Kubelka–Munk formula.

The threshold wavelength is the maximum wavelength of electromagnetic radiation needed to eject electrons from a material’s surface. This threshold wavelength can be used to assess energy variations during electronic transitions within EFCO. Formula 8 specifies the calculation, where A denotes absorption, G is an empirical constant, and λ_S_ represents the threshold wavelength. Additionally, absorption A can be derived from the absorption rate using Formula 9 [67].

(8) $${\left(A/\lambda \right)}^{2}=G\left(1/\lambda -1/\lambda_{s}\right)$$

(9) $$A=\log\left(1/R\right)$$

Figure 8e,f illustrate the threshold wavelength of EFO and the variation in threshold wavelength for EFCO with different Co ion doping levels. Detailed data can be found in Figure S7. The inverse of the threshold wavelength was determined by extrapolating the linear portion and intersecting it with the X-axis. As the Co ion doping levels increased, the threshold wavelength also increased. This indicated a decrease in the energy required for electronic transitions. This trend aligned with the bandgap change estimated using the Kubelka–Munk formula. Consequently, Co ion doping enhanced the electronic transitions within EFCO in a favorable direction.

When EFCO was exposed to laser irradiation, the d-degenerate energy levels within EFCO split into a lower-energy dt_2g_ level and a higher-energy de_g_ level due to the crystal field effect. This splitting resulted in a combined electronic transition mode. This mode primarily consisted of crystal field transitions between the d-orbitals of Fe ions and transitions of Er ions from the ground state ^4^I_15/2_ to various excited states. The introduction of Co ions significantly modified the band structure of EFCO. The incorporation of Co ions promoted electronic transitions and increased the number of oxygen vacancies and defects in the material. These changes enhanced the transition mechanisms and improved the laser suppression performance. Additionally, the increase in threshold wavelength reduced the energy required for electronic transitions, further facilitating these transitions. Consequently, the variation in the band gap induced by Co ions affected the optical properties of EFCO.

**Figure 9:**
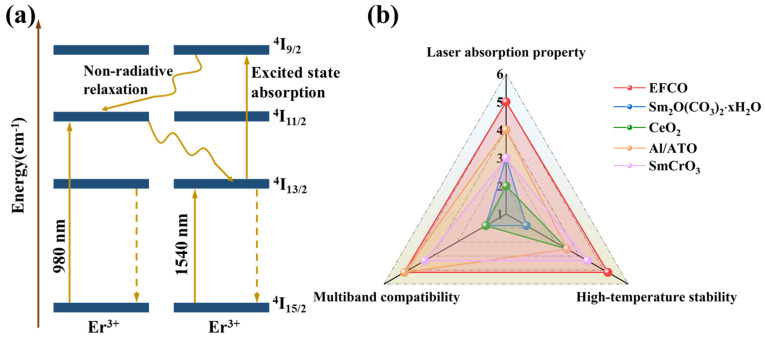
(**a**) The laser suppression mechanism of EFCO. (**b**) The radar chart shows the comparison between our EFCO and other laser suppression materials.

The results indicated that EFCO exhibited high-temperature stability, dual-band compatibility, and laser absorption capabilities. Previous studies have identified various laser suppression materials, including Sm_2_O(CO_3_)_2_⋅xH_2_O [26], CeO_2_ [68], Al/ATO [25], and SmCrO_3_ [69], as illustrated in Figure 9b. However, none of these materials combined all three properties: dual-band compatibility, laser absorption, and high-temperature stability. This unique combination in EFCO underscores its potential for dual-band laser suppression applications.

## 4. Conclusions

In this study, ErFe_0.95_Co_0.05_O_3_ were synthesized by introducing Co^3+^ ions, which have excellent dual-band laser suppression properties, with characteristic absorption in both the 1064 nm and 1540 nm bands, enabling them to effectively resist the risk of combined multiband laser detection. The introduction of Co^3+^ ions simultaneously altered the bandgap of EFCO and enhanced its internal electron mobility, improving its laser suppression capability and its internal electron hopping, which improved its laser suppression capability. As a result, the synthesized ErFe_0.95_Co_0.05_O_3_ exhibited a reflectivity of 22.16% at 1064 nm and 35.63% at 1540 nm. High-temperature tests at 1100 °C further showed that the EFCO retained 95% and 96% of its original laser absorption at 1064 and 1540 nm, respectively. EFCO’s high-temperature resistance enabled it to meet the demands of complex high-temperature environments. These results demonstrated the potential of this material for a wide range of applications in multi-band compatible laser suppression.

## Figures and Tables

**Table 1:** Crystallographic data of EFCO (x = 0–0.07).

ErFe_1−x_Co_x_O_3_	X = 0.00	X = 0.01	X = 0.03	X = 0.05	X = 0.07
a (Å)	5.2648	5.2629	5.2629	5.2609	5.2589
b (Å)	5.5823	5.5810	5.5808	5.5791	5.5781
c (Å)	7.5966	7.5934	7.5923	7.5885	7.5847
V (Å^3^)	223.263	223.03	222.99	222.734	222.492
Fe-O(1) (Å)	2.0043 (10)	2.0033 (5)	2.0030 (5)	2.0021 (4)	2.0011 (3)
Fe-O(2) (Å)	2.0292 (8)	2.0284 (4)	2.0284 (4)	2.0277 (4)	2.0273 (4)
Fe-O(1)-Fe (°)	142.752 (3)	142.747 (12)	142.741 (11)	142.737 (10)	142.732 (8)
Fe-O(2)-Fe (°)	144.231 (11)	144.234 (5)	144.237 (5)	144.239 (5)	144.243 (4)
*d*	0.595	0.598	0.598	0.600	0.610
*S*	0.0585	0.05866	0.05863	0.0587	0.0589
R_wp_ (%)	7.218	5.379	5.968	4.466	4.157

**Table 2:** Raman mode classification of EFCO (x = 0.05).

Raman Shift (cm^−1^)	Atomic Motions in EFCO	Mode
116	Er^3+^-O^2−^ vibrations	A_g_^(1)^
141	Er^3+^-O^2−^ vibrations	A_g_^(2)^
163	Er^3+^-O^2−^ vibrations	B_2g_^(2)^
263	Co-rotations of FeO_6_ octahedra	B_1g_^(2)^
332	Rotation of FeO_6_ octahedra around the y-axis	A_g_^(4)^
429	Rotation of FeO_6_ octahedra around the z-axis	A_g_^(5)^
501	Scissor-like bending of O(1)-Fe-O(2) [51]	A_g_^(7)^

## Data Availability

The data used to support the findings of this study are included within the article.

## Supplementary Materials

The following supporting information can be downloaded at: https://www.mdpi.com/article/10.3390/ma18081861/s1. Table S1. Er 4d, Fe 2p, Co 2p, and O 1s fitting results. Figure S1. (a) Fe 2p, (b) Co 2p< (c) Er 4d, and (c) O 1s high-resolution XPS spectra and fitting data. Figure S2. Grain size distribution of EFCO (x = 0, 0.01, 0.03, 0.05, and 0.07). Figure S3. Percentage of EDS element distribution. Figure S4. (a) Comparison of laser inhibition performance of EFCO (x = 0.05) at 1100 °C with different oxidation times. (b) Comparison of detailed reflectance of EFCO (x = 0.05) at 1064 nm and 1540 nm with different oxidation times. Figure S5. Detailed data of band gap with different doping contents. Figure S6. Detailed data of valence band spectra for different doping contents. Figure S7. Detailed data of threshold wavelengths for different doping contents.
