# Supplementary material for: Co-Doped ErFeO3 for Dual-Band Laser Absorption with High-Temperature Stability
Source: Materials (Basel). 2025 Apr 18;18(8):1861. doi: 10.3390/ma18081861 (PMC12028810; doi:10.3390/ma18081861)
Supplement: Supplementary file 1 [file materials-18-01861-s001.zip › materials-3566342-supplementary.pdf]

Co-doped $ErFeO_3$ for Dual-Band Laser Absorption with High-Temperature Stability

Table S1. Er 4d, Fe 2p, Co 2p and O 1s fitting results.

	Peak	FWHM(eV)	Aera
Er^{3+} 4d	167.58	3.63	53158.88
Fe^{3+} $2p_{2/3}$	710.3	2.89	49875.14
Fe^{4+} $2p_{2/3}$	712.36	3.59	22693.22
Fe^{3+} $2p_{1/2}$	724.6	3.52	32129.76
Fe $2p_{2/3}$ sat.	717.77	3.53	10409.59
Fe $2p_{1/2}$ sat.	731.99	2.94	2636.21
Co^{3+} $2p_{3/2}$	780.58	3.51	14702.86
Co^{2+} $2p_{3/2}$	782.82	3.42	6717.88
Co $2p_{1/2}$	795.88	2.71	3144.57
Co $2p_{3/2}$ sat.	785.69	2.73	5419.05
Co $2p_{3/2}$ sat.	787.89	2.18	7318.22
Co $2p_{1/2}$ sat.	804.99	2.67	2607.17
O 1S	528.78	1.91	88583.47
V_{OS}	531.02	2.62	67178.96

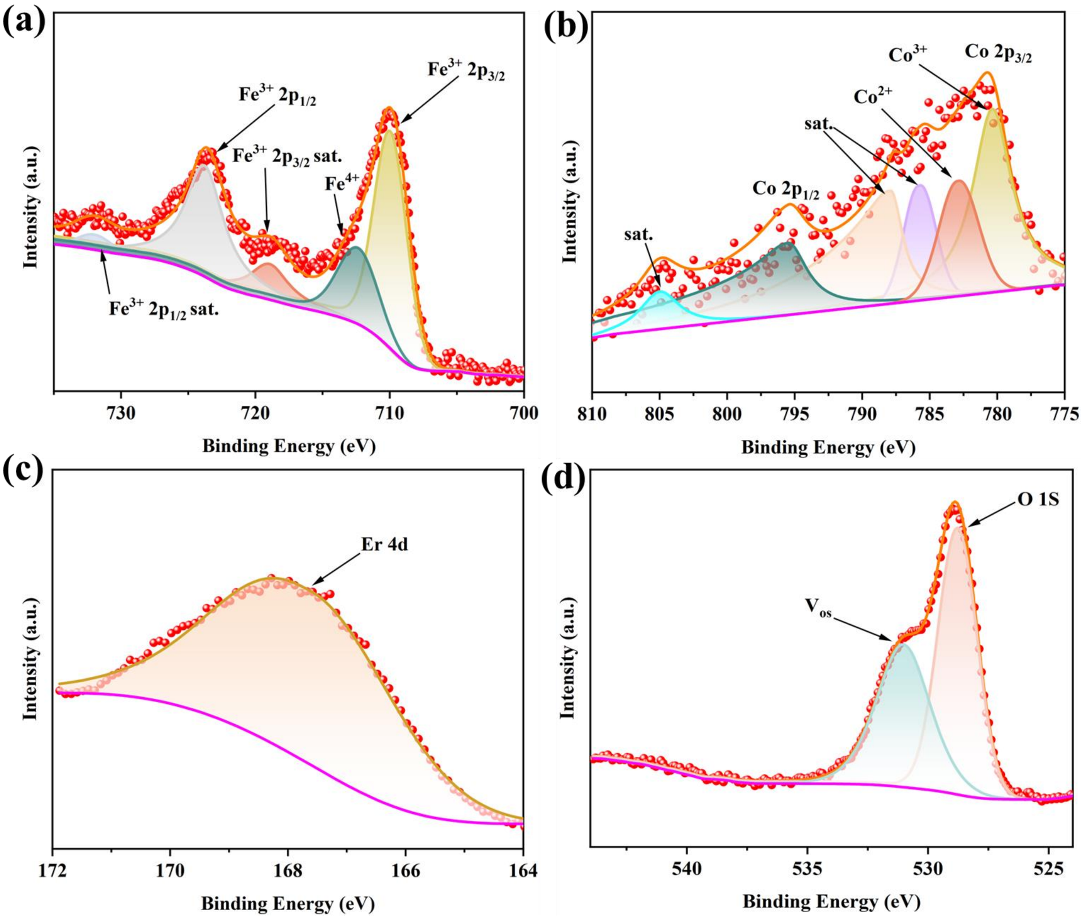

Figure S1. (a) Fe 2p; (b) Co 2p; (c) Er 4d; (c) O 1s high-resolution XPS spectra and fitting data.

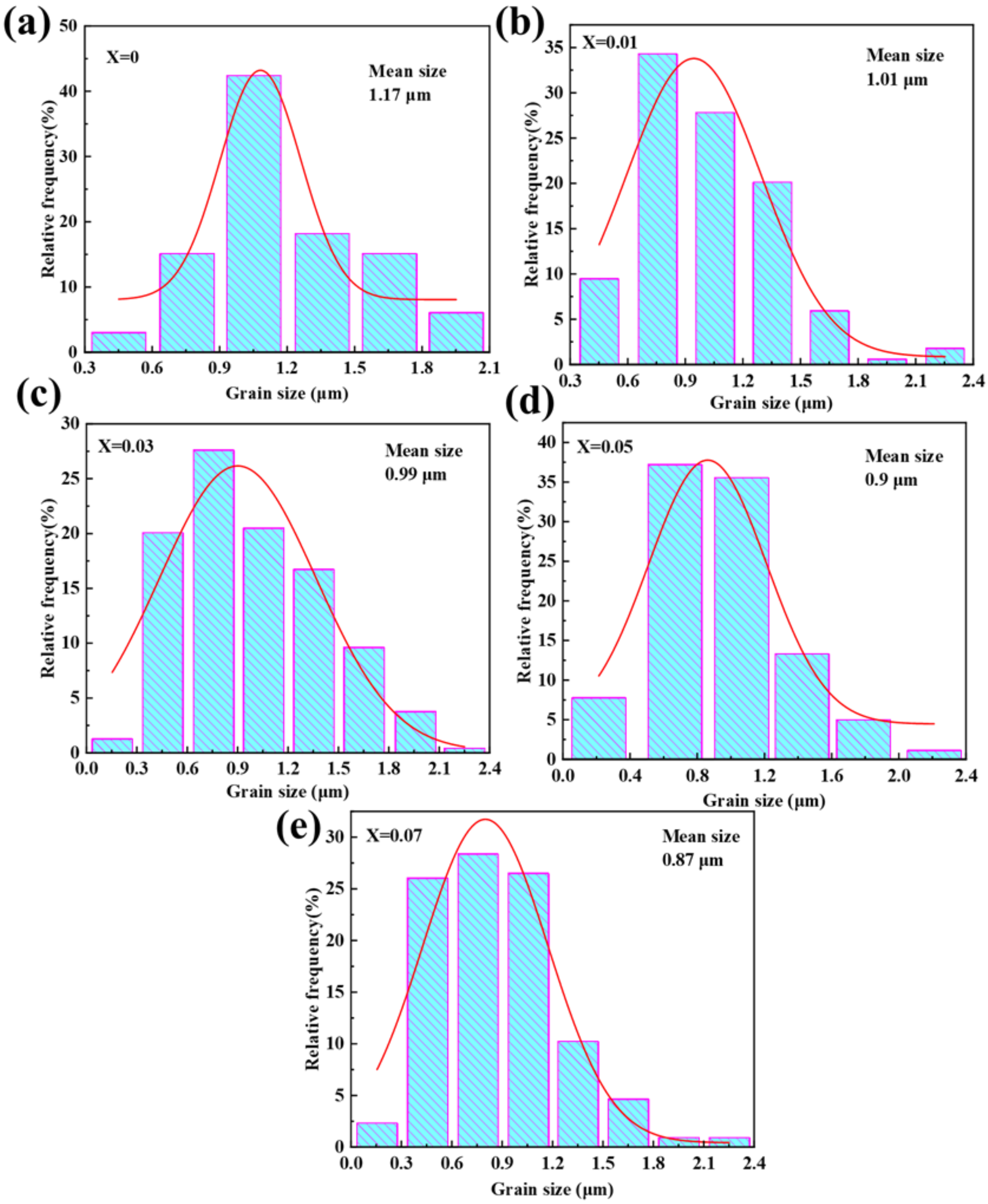

Figure S2. Grain size distribution of EFCO (x=0, 0.01, 0.03, 0.05, 0.07).

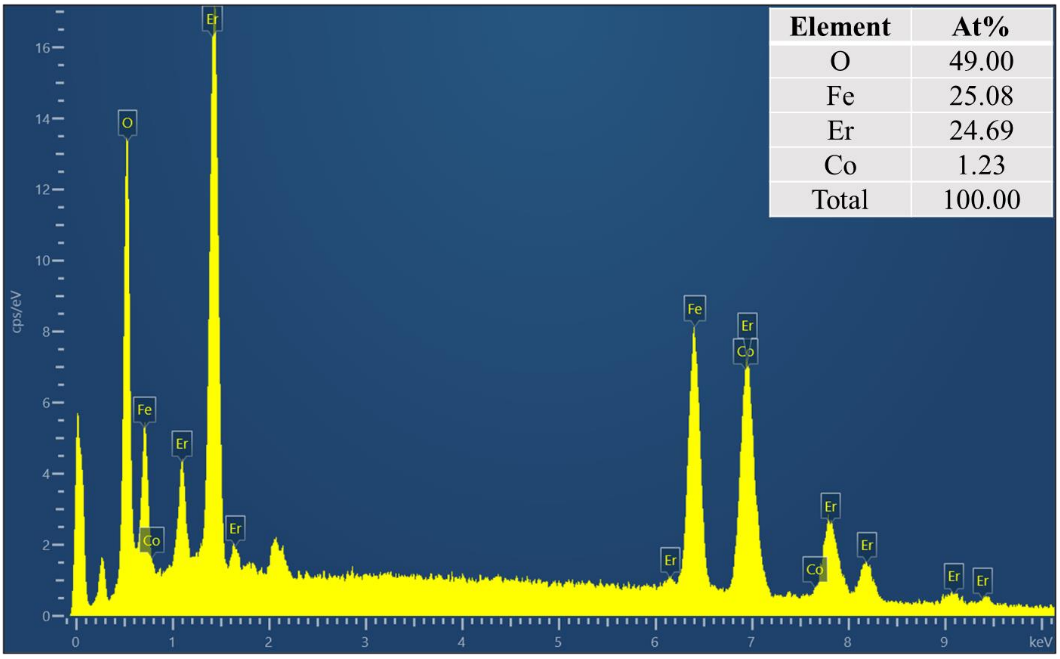

Figure S3. Percentage of EDS element distribution.

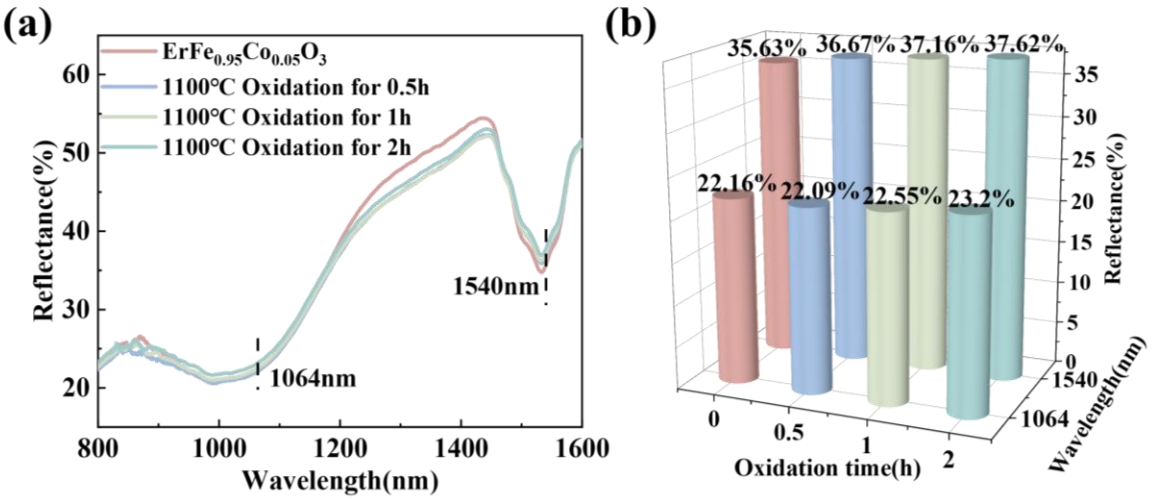

Figure S4. (a) Comparison of laser inhibition performance of EFCO (x=0.05) at 1100 °C with different oxidation times; (b) Comparison of detailed reflectance of EFCO (x=0.05) at 1064 nm and 1540 nm with different oxidation times.

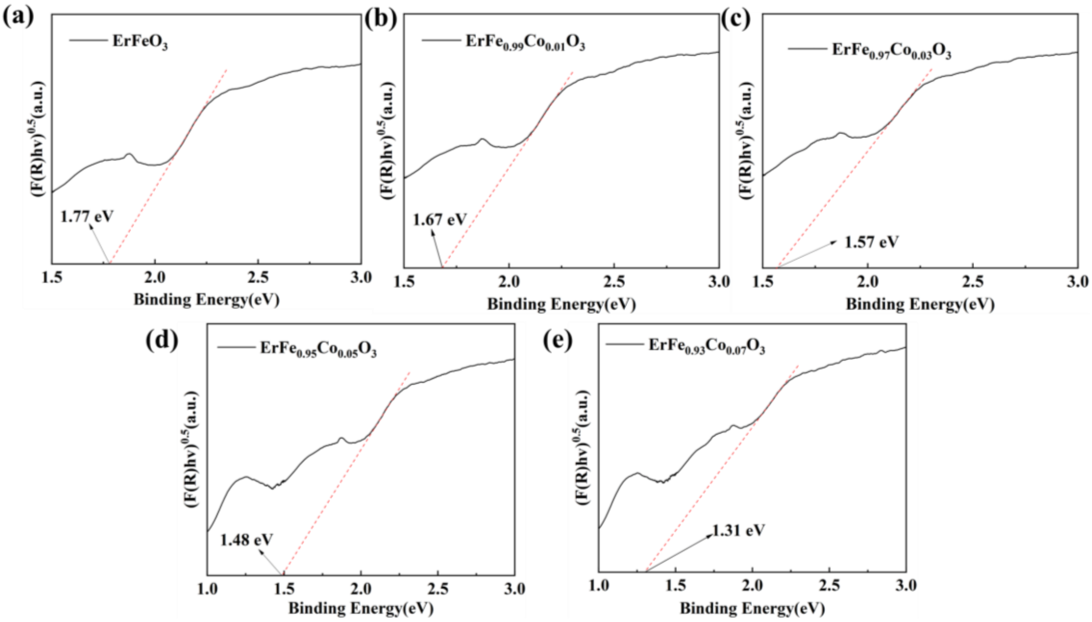

Figure S5. Detailed data of band gap with different doping contents.

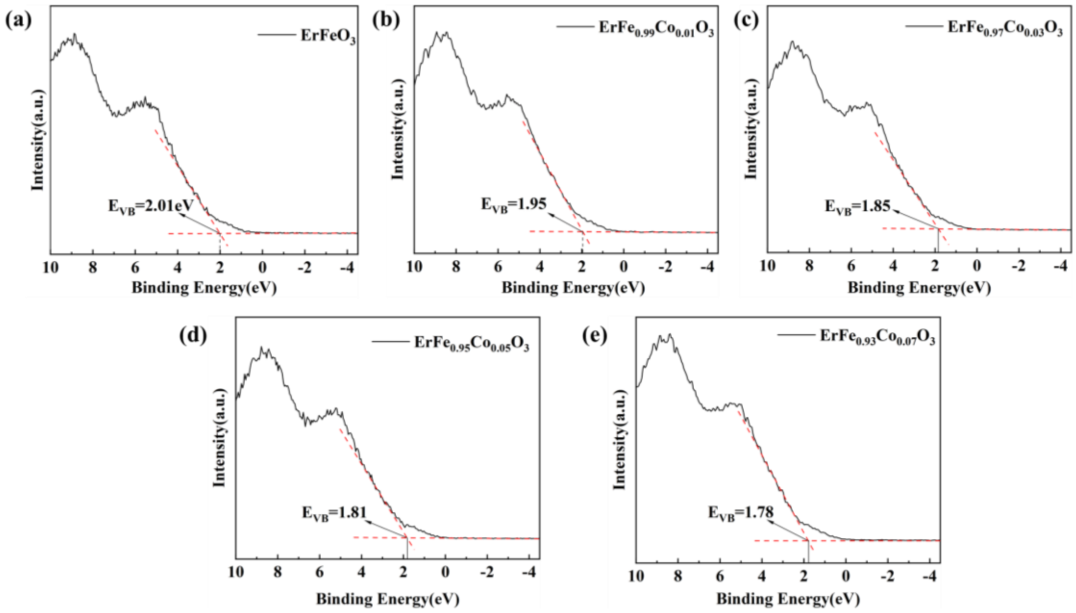

Figure S6. Detailed data of valence band spectra for different doping contents.

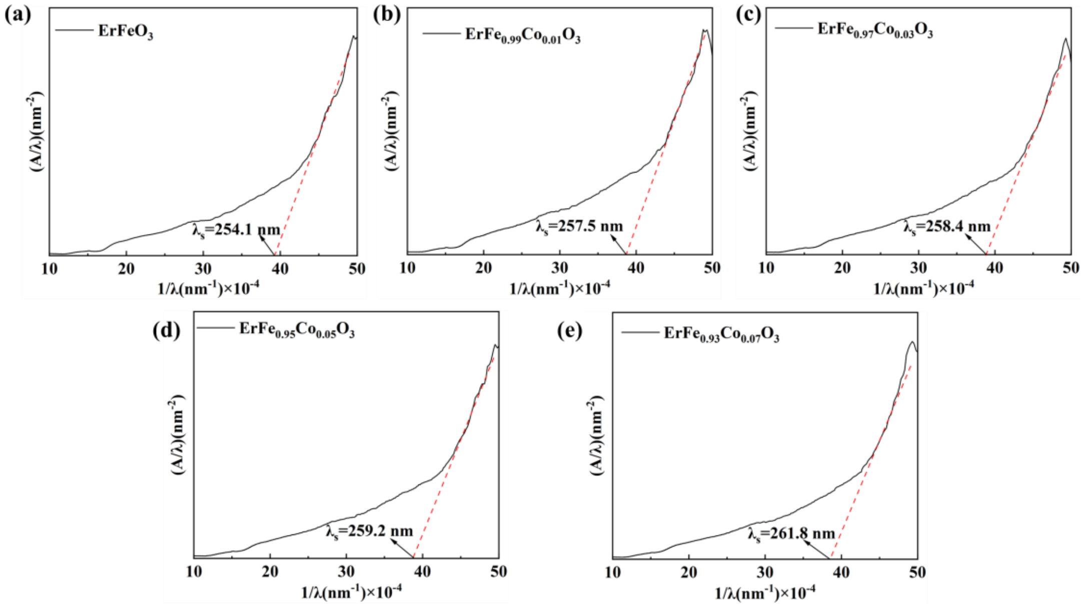

Figure S7. Detailed data of threshold wavelengths for different doping contents.
